# The incidence of chronic drug-induced liver injury: a systematic review and meta-analysis

**DOI:** 10.3389/fphar.2026.1710414

**Published:** 2026-01-29

**Authors:** Yu Zhang, Yu-Lin Ren, Xiang-Rui Song, Xi-Jie He, Xiao-Yu Wen

**Affiliations:** 1 Department of Hepatology, Center of Infectious Diseases and Pathogen Biology, The First Hospital of Jilin University, Changchun, Jilin, China; 2 Department of Epidemiology and Biostatistics, School of Public Health, Jilin University, Changchun, Jilin, China

**Keywords:** chronic, drug-induced liver injury, incidence, meta-analysis, systematic review

## Abstract

**Objectives:**

Drug-induced liver injury (DILI) is a recognized adverse drug event. Although most cases present with acute hepatic damage, evidence indicates that a proportion progress to persistent liver injury. The absence of a standardized definition for chronic DILI has contributed to significant discrepancies in reported incidence rates across clinical studies. This meta-analysis aims to determine the pooled incidence of chronic DILI, providing robust epidemiological evidence.

**Methods:**

This meta-analysis was conducted in accordance with the PRISMA and MOOSE guidelines. A systematic search was conducted in PubMed, Web of Science, Embase and Cochrane Library databases from their respective inception dates to 11 July 2025. The quality of cohort studies was assessed using the NOS. A random-effects model was used to calculate the pooled incidence of chronic DILI, expressed as corresponding 95% confidence intervals (*CI*s). Subgroup analyses were performed to explore potential sources of heterogeneity. Publication bias was assessed and sensitivity analyses were conducted. All statistical tests were two-tailed, and a *P* value <0.05 was considered statistically significant.

**Results:**

A total of 24 studies were included in the final analysis. The pooled incidence of chronic DILI (based on a duration of liver injury lasting more than 6 months without distinguishing the suspected drugs) was 14.09% (95% *CI*: 10.35%–18.29%; *I* ^*2*^ = 80.76%). Four studies that reported the incidence of chronic DILI based on a 12-month follow-up (without distinguishing causative drugs) showed a pooled incidence of 7.95% (95% *CI*: 5.16%–11.24%; *I* ^*2*^ = 54.8%). The pooled incidence of chronic DILI attributed to antimicrobial drugs (6-month follow-up) was 14.56% (95% *CI*: 10.86%–18.65%; *I* ^*2*^ = 0%).

**Conclusion:**

Chronic DILI accounts for a clinically certain proportion of DILI cases. Greater emphasis should be placed on the long-term management and follow-up of patients with DILI to mitigate the risk of chronic progression.

**Systematic Review Registration:**

https://inplasy.com, identifier INPLASY202580021.

## 1 Introduction

Drug-induced liver injury (DILI) refers to the liver injury caused by various prescription drugs, over-the-counter drugs, their metabolites, and other related substances (Shen et al., 2019; Yu et al., 2017). It is one of the more common adverse drug reactions, and its incidence is increasing annually, with regional variations. Clinically, it is mostly acute liver damage; however, some cases persist, resulting in chronic liver injury. The definition of chronic DILI remains controversial and has not yet reached complete uniformity (Hayashi and Fontana, 2014). Most studies define chronic DILI as the presence of liver injury (biochemical, imaging, or histological evidence indicating liver injury or portal hypertension) that persists for more than 6 months after DILI onset (Fontana et al., 2009; Fontana et al., 2014; Yu et al., 2017; Shen et al., 2019). The reported incidence of chronic DILI varies considerably across studies. This controversy over its definition reflects the underlying complexity of chronic DILI. Understanding its epidemiology and influencing factors is of great significance in preventing disease progression. Currently, a comprehensive assessment of the incidence of chronic DILI is lacking. This study therefore aims to estimate the incidence of chronic DILI using a meta-analysis to provide relatively reliable epidemiological data.

## 2 Methods

### 2.1 Search strategy and selection criteria

This systematic review and meta-analysis followed a pre-registered protocol on the International Platform of Registered Systematic Review and Meta-analysis Protocols (INPLASY; registration number: INPLASY202580021; https://inplasy.com), ensuring transparency and adherence to established guidelines. Reporting followed the Preferred Reporting Items for Systematic Review and Meta-Analysis (PRISMA) (Page et al., 2021) statement and Meta-Analysis of Observational Studies in Epidemiology (MOOSE) (Stroup et al., 2000). Three researchers (YZ, Y-LR, and X-RS) conducted searches in PubMed, Web of Science, Embase and Cochrane Library databases from their respective inception dates to 11 July 2025 using the following search terms: (“Chemical and Drug Induced Liver Injury” OR “Hepatitis, Toxic” OR “Drug-Induced Liver Disease” OR “DILI” OR “drug-induced hepatotoxicity” OR “drug-induced liver damage” OR “drug-induced chronic liver failure” OR “idiosyncratic drug-induced liver injury”) AND (“Incidence” OR “Epidemiology” OR “frequency” OR “Follow-Up Studies” OR “Cohort Studies” OR “Prospective Studies” OR “Retrospective Studies”). No limits were applied to the search.

Titles and abstracts of relevant publications were independently screened by the same three researchers, who then reviewed the full texts of potential articles for eligibility. All selected articles met the following inclusion criteria according to the PICOS acronym: Participants (*P*): Patients diagnosed with DILI; Intervention (*I*): not applicable; Comparison (*C*): not applicable; Outcome (*O*): incidence of chronic DILI or relevant data from which the incidence of chronic DILI could be estimated; Study design (*S*): cohort studies with accessible data, published in a peer-reviewed, English-language journal. Exclusion criteria encompassed studies where DILI was attributed to non-drug-related factors and those with a follow-up duration of less than 6 months. If multiple studies originated from the same data network with overlapping periods, only the study with the largest sample size was included to avoid duplication of data. To ensure accuracy, any disagreements that arose during the study selection process were resolved by consensus among the three independent researchers or through discussion with the corresponding author (X-YW).

### 2.2 Data extraction and quality assessment

Three researchers independently extracted the following information from each included study: study details (first author, publication year and country), study characteristics (survey time and study design), and participant information (initial sample size, number of patients at different follow-up times in DILI and chronic DILI, mean age, proportion of males, implicated drugs and clinical types of chronic DILI).

The quality of cohort studies was assessed using the Newcastle–Ottawa Scale (NOS) (Peterson et al., 2011), which evaluates three domains: selection of study groups, comparability of groups, and ascertainment of exposure or outcome. The NOS utilizes a star-based scoring system, with higher scores indicating better quality. Studies scoring 0–3, 4–6, and 7–9 stars were classified as low, medium, and high quality, respectively. Any discrepancies or uncertainties in the quality assessments were resolved through consensus or by discussion with the corresponding author (X-YW).

### 2.3 Statistical analysis

Statistical analyses were performed using R software (version 4.5.1) (R Core Team, 2024). The meta-analysis was conducted with the meta package (8.2–0) in R. A random-effects model was applied to estimate the pooled incidence of chronic DILI and the corresponding 95% confidence intervals (*CI*s). Heterogeneity across studies was assessed using the *I* ^*2*^ statistic, with *I* ^*2*^ > 50% indicating substantial heterogeneity (Higgins et al., 2003). To explore potential sources of heterogeneity, subgroup analyses and meta-regression analyses were performed, as appropriate. Subgroup analyses were categorized by the income level of the country, sample size, study type, and quality assessment. Meta-regression analysis was performed when more than 10 studies were available for analysis.

Publication bias was assessed using funnel plots and Egger’s test (Egger et al., 1997). Sensitivity analyses were conducted by sequentially excluding individual studies to evaluate the robustness of the pooled estimates. All statistical tests were two-tailed, and a *P* value <0.05 was considered statistically significant.

## 3 Results

### 3.1 Identification and description of studies

**FIGURE 1:**
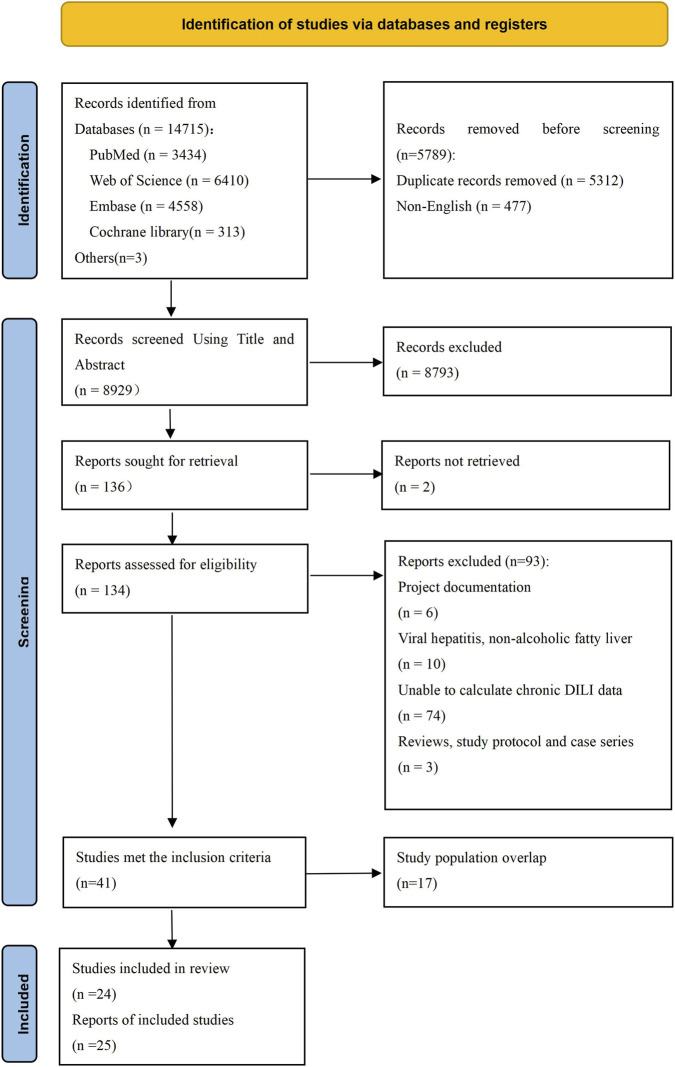
The flow diagram of study selection.

A total of 8,929 articles were initially identified, of which 41 studies met the inclusion criteria after screening; 17 studies were excluded due to overlapping study populations. Ultimately, 24 studies were included in the study. (Björnsson et al., 2013; Ghabril et al., 2013; Russo et al., 2014; Alqahtani et al., 2015; Chalasani et al., 2015; deLemos et al., 2016; Kuzu et al., 2016; Zhu et al., 2016; Shen et al., 2019; Zhu et al., 2020; Tian et al., 2021; Stephens et al., 2021; Koshy et al., 2022; Li et al., 2022; Zeng et al., 2022; Chalasani et al., 2023; Fontana et al., 2023; Bonkovsky et al., 2024; Conlon et al., 2024; Fontana et al., 2024; Pop et al., 2024; Ahmad et al., 2025; Gopalakrishna et al., 2025; Bessone et al., 2025). All patients diagnosed with DILI were recruited from medical institutions across 24 studies. Among these studies, 10 articles did not distinguish the causative drugs. Of these 10 studies, seven articles defined chronic DILI based on a 6-month follow-up period, one of which also reported outcomes at 12 months. The remaining three studies defined chronic DILI based on a 12-month follow-up period. Of the 24 studies, 14 articles identified the suspected drugs causing chronic DILI, among which antimicrobials were the most common drugs (n = 6). The PRISMA flow diagram is presented in Figure 1. The followed-up sample sizes of the included studies ranged from 6 to 25,927, with the proportion of males varying between 21.21% and 83.33%. Study quality assessment scores ranged from 6 to 9, with 11 studies (45.83%) classified as high quality and 13 studies (54.17%) as moderate quality. A detailed summary of study characteristics is provided in Table 1 and Supplementary Table S1.

**TABLE 1:** Characteristics of studies included in this meta-analysis.

No.	First author and publication year	Country	Survey time	Study design	No. of total subjects (DILI)	No. of DILI followed up	
						6 months	12 months
1	Koshy et al. (2022)	India	2006–2016	Prospective	143	—	66
2	Zhu et al. (2020)	China	2009–2012	Retrospective	140	140	—
3	Björnsson et al., (2013)	Iceland	2010–2012	Prospective	96	96	—
4	Shen et al. (2019)	China	2012–2014	Retrospective	25,927	25,927	—
5	Li et al. (2022)	China	2020–2022	Retrospective	224	224	—
6	Tian et al. (2021)	China	2018–2019	Prospective	95	95	95
7	Kuzu et al. (2016)	Turkey	2008–2013	Retrospective	87	82	—
8	Chalasani et al. (2015)	United States	2004–2013	Prospective	899	720	—
9	Stephens et al., (2021)	Spain	1994–2018	Prospective	843	—	625
10	Bessone et al., (2025)	Latin American countries	2011–2022	Prospective	468	—	206
11	Ahmad et al. (2025)	United States	2004–2022	Prospective	61	45	—
12	Alqahtani et al. (2015)	United States	2004–2012	Prospective	19	19	—
13	Russo et al. (2014)	United States	2004–2012	Prospective	22	22	—
14	Fontana et al. (2023)	United States	2004–2020	Prospective and retrospective	93	70	—
15	Pop et al. (2024)	United States	2003–2021	Prospective and retrospective	10	6	—
16	deLemos et al. (2016)	United States	2004–2014	Prospective	117	110	—
17	Zeng et al. (2022)	China	2014–2018	Retrospective	122	122	—
18	Zhu et al. (2016)	China	2009–2014	Retrospective	1985	563	—
19	Conlon et al. (2024)	United States	2004–2022	Retrospective	30	30	—
20	Chalasani et al. (2023)	United States	2004–2021	Prospective and retrospective	78	67	—
21	Bonkovsky et al. (2024)	United States	2004–2022	Prospective and retrospective	55	48	—
22	Ghabril et al. (2013)	United States	2004–2010	Prospective	32	32	—
23	Gopalakrishna et al. (2025)	United States	2004–2024	Prospective	13	11	—
24	Fontana et al. (2024)	United States	2021–2023	Prospective and retrospective	23	16	—

No. of chronic DILI		Basic population information in chronic DILI		Causes leading to DILI	Clinical subtypes of chronic DILI			Quality assessment Score
6 months	12 months	Male gender proportion (%)	Age (years)		Hepatocellular injury (%)	Cholestatic injury (%)	Mixed injury (%)	
—	2	—	—	Undistinguished	0.00	100.00	0.00	6
33	—	21.21	51.2 ± 17.7	Undistinguished	72.73	6.06	21.21	9
7	—	—	—	Undistinguished	—	—	—	6
3,371	—	—	—	Undistinguished	—	—	—	6
19	—	73.68	25–78	Undistinguished	63.16	26.32	10.53	6
16	9	—	—	Undistinguished	—	—	—	7
12	—	—	—	Undistinguished	50.00	33.33	16.67	7
126	—	—	—	Undistinguished	—	—	—	8
—	45	—	—	Undistinguished	—	—	—	6
—	24	—	—	Undistinguished	50.00	37.50	12.50	6
6	—	—	—	Fluoroquinolone	—	—	—	6
3	—	—	49–67	Cefazolin	33.33	0	66.67	7
4	—	—	—	Statin	75.00	25.00	0	6
14	—	50	—	Trimethoprim-sulfamethoxazole	21.43	42.86	35.71	6
1	—	—	—	Amiodarone	—	—	—	6
12	—	83.33	32–72	Amoxicillin-clavulanate	16.67	33.33	50.0	6
39	—	—	—	Chinese herbal medicine	—	—	—	7
70	—	—	—	Chinese herbal medicine	—	—	—	7
5	—	—	—	Azithromycin	—	—	—	7
11	—	—	—	Nitrofurantoin	—	—	—	8
9	—	—	—	Non-steroidal anti-inflammatory drugs	55.56	33.33	11.11	8
4	—	—	—	Intravenously administered medications	—	—	—	6
0	—	—	—	Medications for alcohol use disorder	—	—	—	7
2	—	—	—	COVID-19 mRNA vaccination	*—*	—	—	6

### 3.2 Incidence of chronic DILI

**FIGURE 2:**
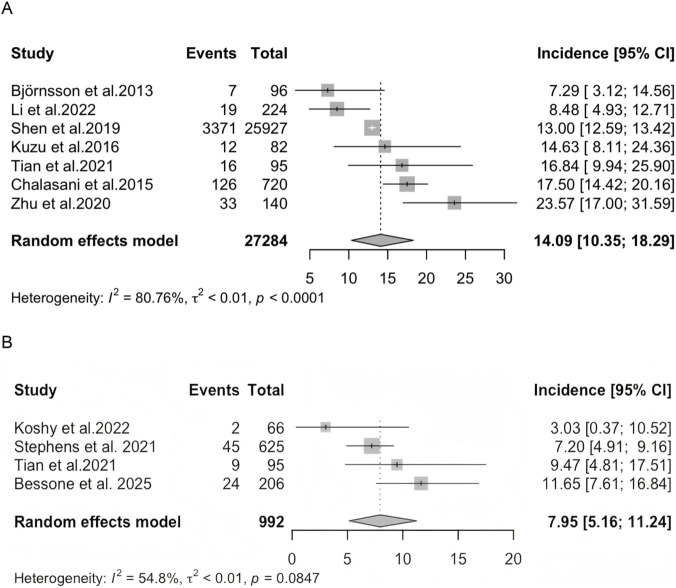
The incidence of chronic DILI without distinguishing the causing drug among patients with DILI. **(A)** Chronic DILI was defined by a 6-month follow-up period). **(B)** Chronic DILI was defined by a 12-month follow-up period).

Based on a duration of liver injury lasting more than 6 months, seven studies reported the incidence of chronic DILI without distinguishing the suspected drugs. The pooled incidence from these studies was 14.09% (95% *CI*: 10.35%–18.29%; *I* ^*2*^ = 80.76%) (Figure 2A). When a 12-month follow-up period was applied, four studies reported the incidence of chronic DILI without distinguishing the suspected drugs. The pooled incidence was 7.95% (95% *CI*: 5.16%–11.24%; *I* ^*2*^ = 54.8%) (Figure 2B).

Among the seven studies on chronic DILI defined using a 6-month follow-up period, three articles reported the clinical patterns of liver injury (hepatocellular injury, cholestatic injury, or mixed injury, based on the “R” ratio (Yu et al., 2017; Fontana et al., 2009)). The most commonly clinical pattern of chronic DILI was hepatocellular injury. In each of these studies, the proportion of hepatocellular type was greater than 50% or more (Kuzu et al., 2016; Zhu et al., 2020; Li et al., 2022).

**FIGURE 3:**
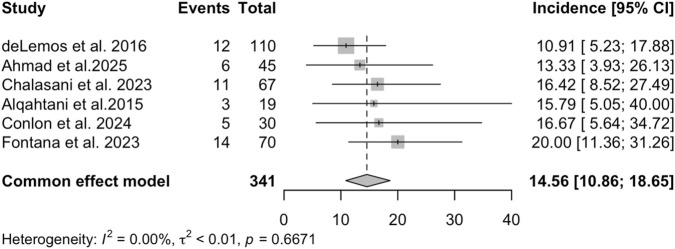
The incidence of chronic DILI induced by antimicrobial drugs (defined by a 6-month follow-up period) among patients with DILI.

A total of 14 studies identified the specific causes of chronic DILI. In six of these studies, chronic DILI was caused by antimicrobial drugs. Figure 3 presents the pooled incidence of chronic DILI (defined by a 6-month follow-up period) caused by antimicrobial drugs, which was 14.56% (95% *CI*: 10.86%–18.65%; *I* ^*2*^ = 0%). The other eight studies reported a variety of suspected causes for chronic DILI with the following incidence rates: stains (18.18% (Russo et al., 2014)), amiodarone (16.67% (Pop et al., 2024)), Chinese herbal medicine (31.97% (Zeng et al., 2022) and 12.43% (Zhu et al., 2016)), non-steroidal anti-inflammatory drugs (18.75% (Bonkovsky et al., 2024)), intravenously administered medications (12.50% (Ghabril et al., 2013)), medications for alcohol use disorder (0% (Gopalakrishna et al., 2025)), and COVID-19 mRNA vaccination (12.50% (Fontana et al., 2024)).

### 3.3 Subgroup analyses

The results of the subgroup analyses are presented in Table 2. Study quality (*P* < 0.005) was significantly associated with the incidence of chronic DILI, whereas income level, sample size, and study type showed no significant association (all *P* > 0.05).

**TABLE 2:** Subgroup analyses of the incidence of chronic DILI without distinguishing the suspected drugs (defined by a 6-month follow-up period) among patients with DILI.

Subgroups	Categories	No. of studies	Incidence, 95% CI (%)	*I* ^2^ (%)	*P* values within subgroups	*P* values across subgroups
Income level	Upper-middle-income	5	14.60 [10.02–19.86]	76.5	0.002	0.707
	High-income	2	12.48 [4.39–23.78]	87.0	0.005	​
Sample size	≤200	4	15.30 [9.01–22.84]	75.0	0.007	0.550
	>200	3	13.04 [8.65–18.17]	87.4	<0.001	​
Study type	Retrospective	4	14.24 [8.80–20.69]	80.9	0.001	0.961
	Prospective	3	13.97 [8.14–21.00]	74.3	0.020	​
Quality assessment	High	4	17.94 [15.38–20.64]	14.0	0.322	<0.005
	Moderate	3	10.29 [6.97–14.16]	72.5	0.026	​

### 3.4 Sensitivity analyses and publication bias

Sensitivity analyses did not find outlying studies that significantly changed primary results after removal (Supplementary Figure S1). Egger’s test showed no significant evidence of publication bias (*t* = 0.72, *P* = 0.5053) (Supplementary Figure S2).

## 4 Discussion

The definition of chronic DILI has not yet been standardized, which poses a challenge for obtaining a unified assessment of its incidence. Initially, hepatocellular injury lasting more than 3 months was regarded as chronic DILI (Bénichou, 1990). Globally, chronic DILI has often been defined based on a duration of liver injury lasting more than 6 months (Fontana et al., 2009; Fontana et al., 2014; Chalasani et al., 2015; Yu et al., 2017; Wang et al., 2018); however, a considerable number of studies have defined chronic DILI using a 12-month threshold (Medina-Caliz et al., 2016; European Association for the Study of the Liver, 2019; Koshy et al., 2022). Additionally, some studies have defined chronic DILI as persistent laboratory abnormalities for more than 3 months for hepatocellular injury and more than 6 months for cholestasis and mixed patterns (Andrade et al., 2005; Andrade et al., 2006; Idilman et al., 2010). Our research found that after the identification of DILI, the incidence of chronic DILI (defined by a persistent liver injury for more than 6 months) was 14.09% (95% *CI*: 10.35%–18.29%; *I* ^*2*^ = 80.76%), and the incidence defined by a duration over 12 months was 7.95% (95% *CI*: 5.16%–11.24%; *I* ^*2*^ = 54.8%). This variation highlights that the differences in definitions may influence the reported incidence of chronic DILI. Unifying the temporal definition of chronic DILI as soon as possible is essential to the epidemiological data of chronic DILI. We conducted a subgroup analysis across countries with different income levels, which showed that the incidence of chronic DILI was higher in upper-middle-income countries (14.60%) than in high-income countries (12.48%), although there was no statistical significance. Upper-middle-income countries included China and Turkey, while high-income countries included the United States and Iceland. This suggests that socioeconomic factors may have potential impacts on disease progression, and this discrepancy may also be related to variations in the profiles of suspected hepatotoxic drugs. For example, in the study conducted in mainland China, among the implicated drugs, Traditional Chinese medicine (TCM) or herbal and dietary supplements (HDS) accounted for 26.81%, and anti-infectious agents accounted for 6.08% (Shen et al., 2019). Antimicrobials were the most common causative drugs in the U.S. Drug Induced Liver Injury Network (DILIN) (Chalasani et al., 2015). Many drugs have been associated with the development of chronic DILI, including antimicrobials (Stine and Chalasani, 2015). Our study reported the incidence of chronic DILI caused by antimicrobial drugs was 14.56% (95% *CI*: 10.86%–18.65%; *I* ^*2*^ = 0%). The studies were all sourced from the DILIN (Alqahtani et al., 2015; deLemos et al., 2016; Fontana et al., 2023; Chalasani et al., 2023; Conlon et al., 2024; Ahmad et al., 2025). In our study, two studies reported the incidence of chronic herbal-induced DILI, with notable variability in the reported rates between them (Zhu et al., 2016; Zeng et al., 2022). However, this finding was based on only two individual publications among 24 included studies. Further in-depth research on chronic herbal-induced DILI is required to better understand its prognosis and clinical course.

In the studies that did not differentiate causative drugs and defined chronic DILI as liver injury persisting more than 6 months, we included three articles that described the patterns of chronic liver injury. The main pattern observed was hepatocellular injury, accounting for 50.00%–72.73% (Kuzu et al., 2016; Zhu et al., 2020; Li et al., 2022). In the study by Medina-Caliz et al. (2016), both chronic and acute DILI were mainly characterized by a hepatocellular pattern. This is consistent with the articles we have included. It should be noted that we only calculated the proportion of clinical types in chronic DILI rather than the incidence of progression to chronic DILI among different initial injury types. The limited number of studies precluded meaningful statistical analysis, warranting future validation.

Early identification of chronic DILI remains crucial for preventing adverse outcomes. Established clinical risk factors include advanced age (Fontana et al., 2015; Medina-Caliz et al., 2016; Yu et al., 2024), female sex (Wang et al., 2022), specific racial backgrounds (Chalasani et al., 2017), and pre-existing comorbidities such as liver disease (Yu et al., 2024), diabetes (Yu et al., 2024), heart disease (Fontana et al., 2014), and active malignancy (Fontana et al., 2014). Regarding clinical injury patterns, the hepatocellular type was the most prevalent pattern in chronic DILI in the three studies in our research. This dominant proportion is likely attributable to the high baseline proportion of hepatocellular injury in the overall DILI population, resulting in its substantial representation among chronic cases. In contrast, cholestatic and mixed patterns are associated with significantly longer recovery times and a higher risk of progression to chronicity (Zhu et al., 2020; Fontana et al., 2023; Bonkovsky et al., 2024). A key limitation of the available data in this article is that they allowed for the calculation of injury pattern distribution within chronic DILI cohorts, but not the incidence of chronicity for each initial pattern. Several biochemical parameters are also independent predictors, including dyslipidemia (Medina-Caliz et al., 2016), delayed total bilirubin clearance (Zhu et al., 2020), and elevated alkaline phosphatase levels (Fontana et al., 2014). Other indicators also show promise; for instance, serum taurocholic acid was shown to predict persistent biochemical abnormalities at 6 months in Tian et al.’s study (Tian et al., 2021), while Zeng et al. demonstrated that baseline lymphocyte count and cholinesterase levels were predictive of chronic herb-induced liver injury (Zeng et al., 2022). In He et al.’s study, a metabolomic analysis revealed that dysregulation of lipid metabolism was associated with the progression of DILI-related fibrosis (He et al., 2022). To facilitate early risk stratification, non-invasive prediction models have been developed, such as the clinic-radiomics model (Fu et al., 2023), which distinguishes chronic DILI from recovered patients, the noninvasive prediction nomogram model named BNR-6 score (for the early prediction of biochemical non-resolution of chronic DILI at 12 months) (Wang et al., 2022), and the model of baseline clinicopathological features (prediction of biochemical non-resolution of DILI at 6 months) (Bihari et al., 2025). Nonetheless, evidence supporting the value of biochemical tests and models for chronic DILI remains scarce. Unfortunately, there is a paucity of reports on the risk factors for chronic DILI among the included literature, precluding formal statistical analysis.

There are some limitations in our research. The Chinese study accounted for the vast majority of cases, which may introduce regional representation bias. The heterogeneity of the studies was relatively high, and no source of heterogeneity could be found. Due to the limited number of included studies, meta-regression analysis could not be performed, which may affect the precision of the results. In addition, as this meta-analysis was limited to English studies, it is necessary to confirm the research results in future meta-analyses involving both English and non-English databases. Finally, in the included studies, it was not possible to calculate the proportion of cirrhosis or liver cancer in chronic DILI.

In conclusion, the incidence of DILI varies across countries, yet overall rates continue to rise. Most DILI patients have acute clinical courses, but some patients exhibit persistent liver injury beyond 6 months after onset. In this study, the incidence of chronic DILI (defined by a 6-month follow-up period) was 14.09%. It is essential to understand the incidence of chronic DILI, increase public awareness of its prognosis, and recognize that some cases can progress to chronic disease. To better evaluate the epidemiology of chronic DILI, greater emphasis should be placed on treatment and long-term follow-up.

## Data Availability

The original contributions presented in the study are included in the article/Supplementary Material, further inquiries can be directed to the corresponding author.
